# Periphyton effects on bacterial assemblages and harmful cyanobacterial blooms in a eutrophic freshwater lake: a mesocosm study

**DOI:** 10.1038/s41598-017-08083-x

**Published:** 2017-08-10

**Authors:** Yingshun Cui, Long Jin, So-Ra Ko, Seong-Jun Chun, Hyung-Seok Oh, Chang Soo Lee, Ankita Srivastava, Hee-Mock Oh, Chi-Yong Ahn

**Affiliations:** 1 Cell Factory Research Center, Korea Research Institute of Bioscience and Biotechnology, 125 Gwahak-ro, Yuseong-gu, Daejeon, 34141 Republic of Korea; 2 College of Biology and the Environment, Co-Innovation Centre for Sustainable Forestry in Southern China, Nanjing Forestry University, Nanjing, 210-037 China; 3 Department of Environmental Biotechnology, KRIBB School of Biotechnology, Korea University of Science & Technology (UST), 217 Gajeong-ro, Yuseong-gu, Daejeon, 34113 Korea; 4 Culture Collection Team, Freshwater Bioresources Culture Research Division, Nakdonggang National Institute of Biological Resources, Sangju, 37242 Republic of Korea

## Abstract

Periphyton comprises a broad range of autotrophic and heterotrophic organisms that grow on submerged surfaces in aquatic environments. To investigate the ecological roles of periphyton and their symbiotic bacterial assemblages related to the control of cyanobacterial blooms, mesocosm experiments were performed in a eutrophic lake that is usually infested with harmful cyanobacterial blooms. Our results showed that periphyton, together with their symbionts, reduced Chl-*a* concentrations (up to 94%), improved water clarity and effectively controlled cyanobacterial blooms in the treatment mesocosm. Planktonic bacterial compositions varied greatly in the pre-bloom/bloom/post-bloom periods in both mesocosms and were mainly influenced by total dissolved nitrogen (TDN) concentrations. The phylum *Cyanobacteria* was the major component in the water samples until bloom peak, but it was replaced by *Actinobacteria* in the post-bloom period. However, periphyton niches were dominated by *Alphaproteobacteria* throughout the experiments, *Cyanobacteria* proportion being lower. Overall, the results indicated that periphyton and their unique bacterial partners could effectively compete with cyanobacteria and improve water quality. Their underlying interaction mechanism was also suggested to explain how periphyton and their symbionts can reduce cyanobacterial blooms in eutrophic water.

## Introduction

Cyanobacterial blooms occur more often in freshwater because of eutrophication and global warming. Blooms can reduce water quality with the release of toxins (e.g., microcystin) and odorous compounds^1–3^, thereby causing adverse impacts on aquatic ecosystems and human health with economic and ecological consequences^4^. A variety of methods have been developed to control cyanobacterial blooms in eutrophic lakes, reservoirs and rivers. Nonetheless, no single method has succeeded universally. Since each freshwater system has its own characteristics, such as geographic, climatic, hydrologic, trophic and land uses in the watershed area, only limited control methods can have an effect in each particular water body.

Many studies have been conducted regarding the chemical and physical factors that influence cyanobacterial growth, and the corresponding changes in cyanobacterial composition^5, 6^. In addition, the ubiquitous roles of microbes associated with cyanobacteria have been investigated from the microbial communities attached to algae with respect to their symbiosis mechanisms and nutrient exchanges^7–9^. *Proteobacteria* (e.g., *Roseobacter*, *Sulfitobacter*) and *Bacteroidetes* (e.g., *Flavobacterium*) appeared to be consistently associated with phytoplankton (e.g., diatoms)^10, 11^. Rhizobacteria can enhance algal biomass^12, 13^. *Microcystis*-attached bacterial compositions differed from free-living bacterial communities living in the same aquatic ecosystem. In particular, *Gammaproteobacteria* dominated in the *Microcystis*-attached fraction when the bloom was declining^14^. These findings are important from ecological and biogeochemical points of view, because they provide the basis for understanding the algae-microbe interaction processes in nutrient cycles in aquatic ecosystems.

Periphyton (a complex mixture of attached microalgae and bacteria) have recently been suggested to have the potential to improve algal biomass productivity^15, 16^. In addition, cyanobacterial growth was effectively reduced by periphyton cultivation in field experiments as well as in indoor experiments on a small scale^17^. An attached cultivation system is regarded as a promising alternative to the conventional suspended cultivation in biofuel production^18, 19^ and wastewater treatment^20^, because attached microalgae can overcome several drawbacks of suspended culture systems, such as low areal productivity (due to shallow pond depth and the dark zone), easy contamination and the expensive harvest process^21^. Deeper light penetration further encouraged the growth of the attached microalgae, which otherwise could not out-compete free-living algae under normal turbid conditions^22^.

The underlying mechanisms for the control of cyanobacterial blooms were not only attributed to the competition for nutrients but also to the allelochemical production by periphyton^23^. However, the roles of periphytic bacterial as well as planktonic bacterial populations in cyanobacterial blooms have not yet been investigated. In addition, the effect of bacteria-periphyton interaction on cyanobacterial blooms has not been explored. It could be expected that periphyton growth and the dominance of different species of algae and bacteria in the periphyton would influence the growth and metabolism of planktonic cyanobacteria and bacteria. Understanding these microbial interactions could provide new insights and clues to the development of novel control strategies, which could suppress cyanobacterial growth by manipulating the bacterial community in an environmentally friendly manner. Therefore, the purposes of this study were as follows: 1) to explore the effect of attached periphyton on cyanobacterial blooms, 2) to uncover the changes in the planktonic/periphytic bacterial community compositions and 3) to investigate the potential roles of bacteria in the pre-bloom/bloom/post-bloom periods.

## Results

### Biophysicochemical factors in control and treatment

**Figure 1:**
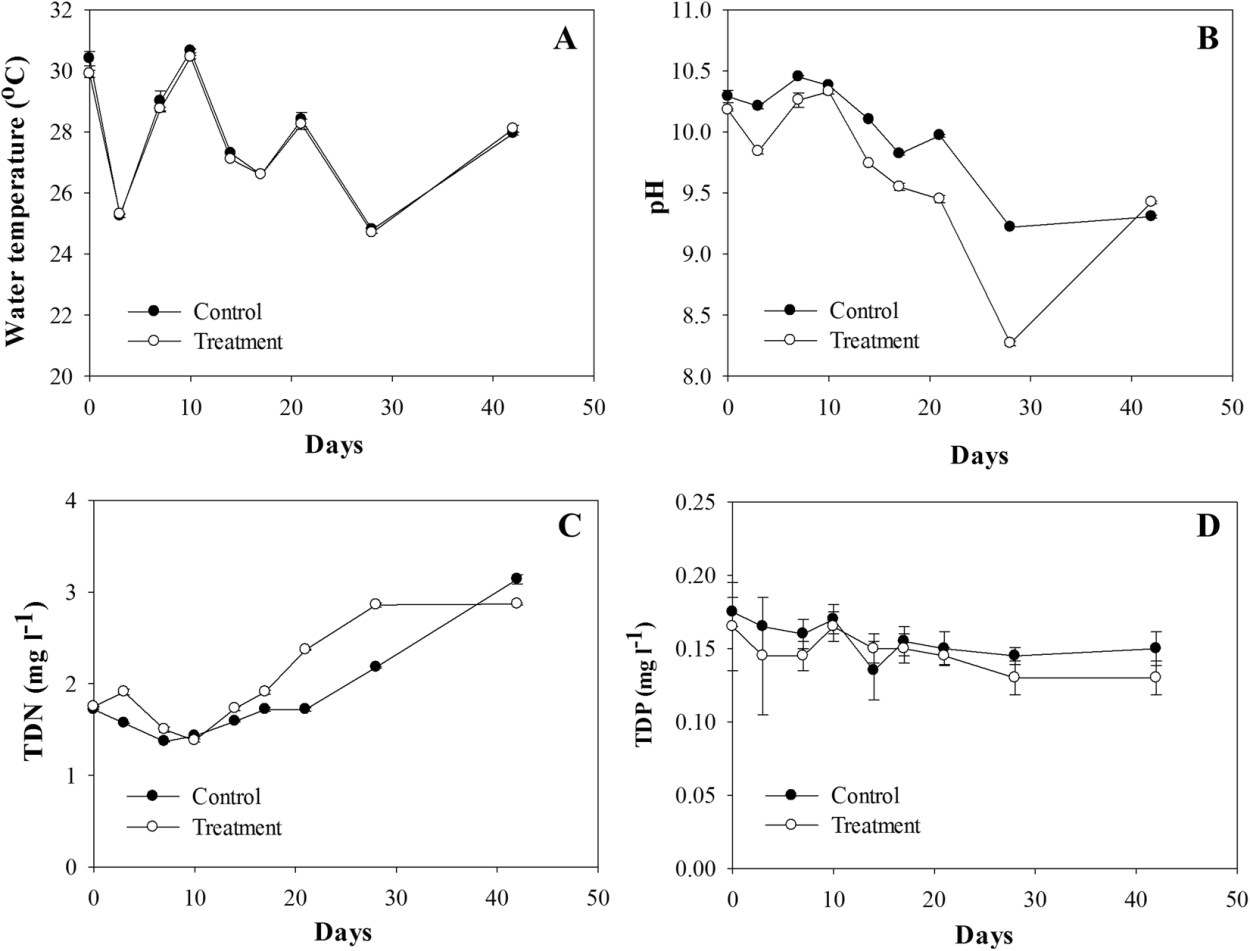
Changes in physicochemical factors in the surface water of the mesocosms: (**A**) water temperature, (**B**) pH, (**C**) concentration of TDN and (**D**) concentration of TDP. Error bars represent standard deviations.

Physicochemical factors such as water temperature, pH, TDN concentration and TDP concentration were generally similar between the control and treatment mesocosms (Fig. 1). The water temperature and pH showed similar patterns in both mesocosms, typically decreasing on rainy days. The pH was consistently higher in the control. TDN concentrations increased gradually in both mesocosms (control: 1.37–3.14 mg l^−1^, treatment: 1.38–2.87 mg l^−1^), with generally higher concentration in the treatment, except for day 42 (control: 3.14 mg l^−1^_,_ treatment: 2.87 mg l^−1^). The TDP concentration decreased slightly (control: 0.14–0.18 mg l^−1^, treatment: 0.13–0.17 mg l^−1^) and was generally higher in the control.

**Figure 2:**
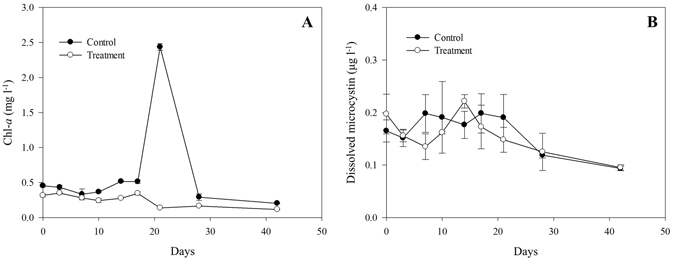
Concentrations of (**A**) Chl-*a* and (**B**) dissolved microcystin in the surface water of the mesocosms. Error bars represent standard deviations.

Compared with T-PL (treatment planktonic samples), Chl-*a* concentrations were up to 18 times higher in the Control (Fig. 2A). A Chl-*a* peak was detected only in the control on day 21 (2.44 mg l^−1^). The surface water of the control mesocosm was much greener than that of the treatment. This difference was more distinct when the surface water was taken in the sampling bottles (Supplementary Fig. S1). Microscopic observations showed that cyanobacteria dominated the surface water of the control and treatment mesocosms, but diverse microalgae were found in the T-PP (treatme﻿nt periphytic sample﻿s) (Supplementary Fig. S2). Microcystin concentrations did not show significant changes or differences between the control and treatment (Fig. 2B). The concentrations remained lower than the drinking water guideline of 1 μg l^−1^ of the World Health Organization^24^. Based on Chl-*a* concentrations in the Control, the three stages were referred to as the pre-bloom period (from day 3 to day 17), bloom period (day 21) and post-bloom period (from day 28 to day 42).

**Figure 3:**
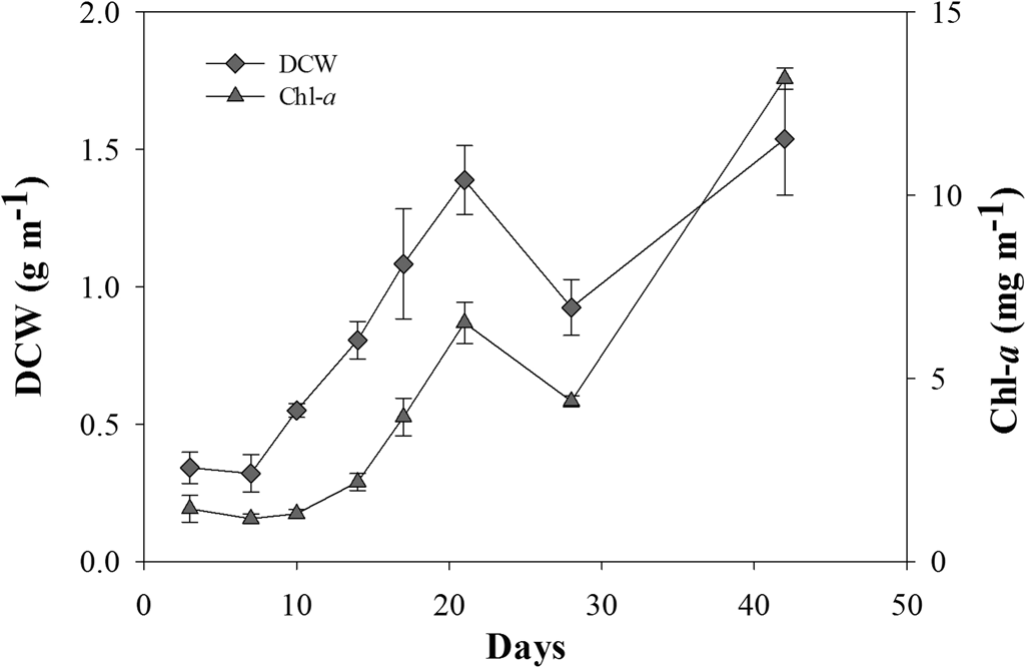
Growth of periphyton biomass, represented as DCW and Chl-*a*. Error bars represent standard deviations.

Periphyton increased exponentially until day 21 (Fig. 3). Periphyton growth was also observed at a depth of 1.5 m but lower than from the surface water. Diatoms and *Scenedesmus* spp. became dominant during this period (Fig. S2C and D). Meanwhile, there was no practical growth of planktonic microalgae in the treatment mesocosm (Fig. 2A). Periphyton growth resumed after a small decrease on day 28 until day 42. The dominant groups of diatoms and *Scenedesmus* spp. were replaced by long filamentous algae, *Oedogonium* spp., at the end of the experiment in the treatment mesocosm (Fig. S3). Free-floating *Oedogonium* spp. flocks were also found in the whole water surface in the treatment mesocosm, but they were restricted to a limited area in the control. The DCW of the total free-floating *Oedogonium* spp. flocks in the treatment were 1.0 kg at day 42. With the dominance of *Oedogonium* spp., the water transparency in the treatment mesocosm was enhanced, in contrast to the turbid control.

### Bacterial community dynamics during mesocosm experiment

A total of 150,170 sequences were obtained from pyrosequencing after removing singletons, low-quality sequences and sequencing contaminants. These sequences were divided into 1,157 different OTUs (97% identity), and a maximum of 231 different OTUs were obtained from the mesocosm experiment.

**Figure 4:**
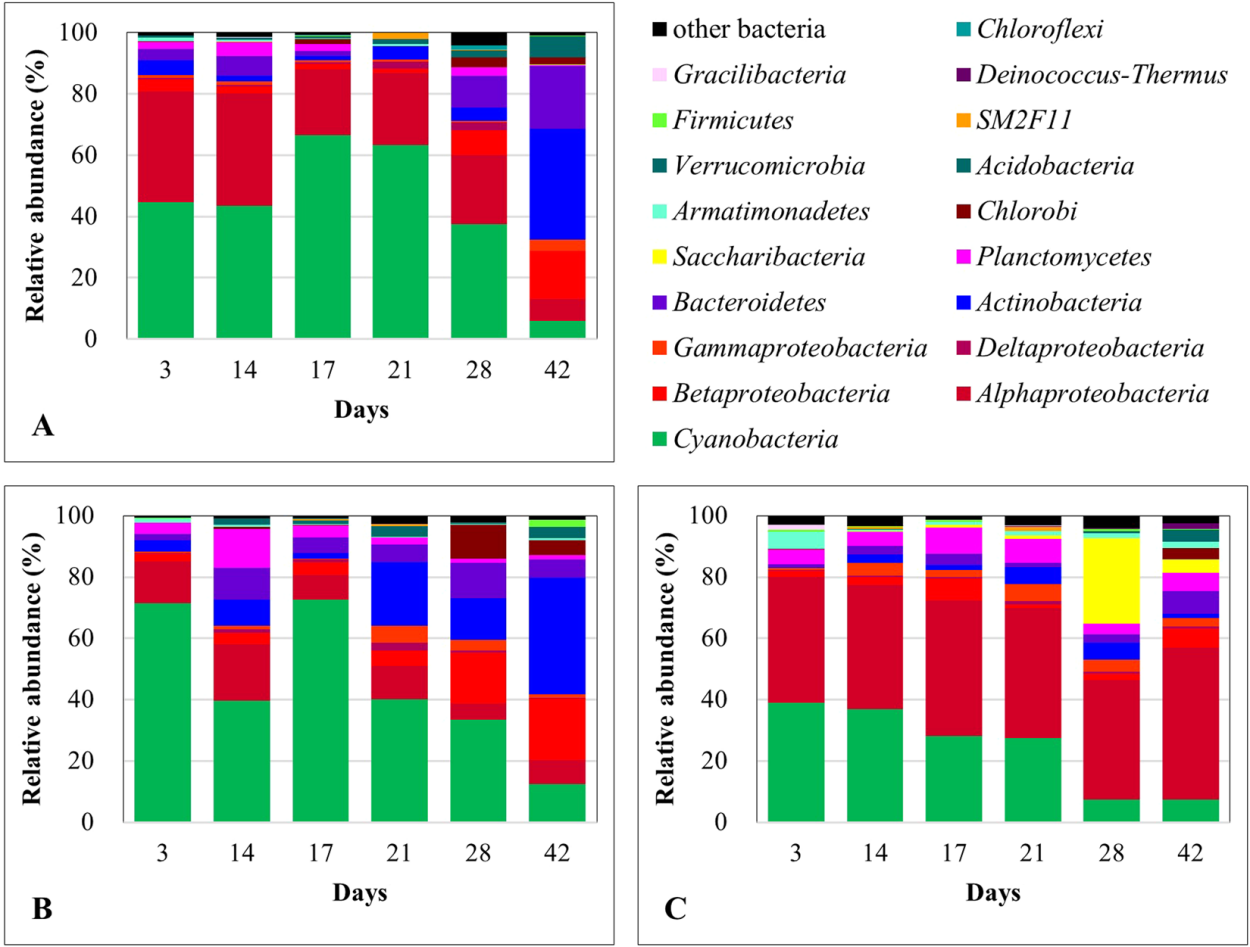
Bacterial community composition in Control, T-PL and T-PP samples during the mesocosm experiment. Phyla are listed in this figure. *Proteobacteria* were separated into *Alphaproteobacteria*, *Betaproteobacteria*, *Gammaproteobacteria* and *Deltaproteobacteria*. (**A**) Control, (**B**) T-PL and (**C**) T-PP samples.

The bacterial communities were dominated by *Cyanobacteria* (Control: 44 ± 22%, T-PL: 45 ± 23% and T-PP: 24 ± 14%) and *Proteobacteria* (Control: 33 ± 8%, T-PL: 23 ± 6% and T-PP: 51 ± 6%) phyla, except for a few samples from the post-bloom period (Fig. 4). Among *Proteobacteria*, *Alphaproteobacteria* was the dominant group. This group constantly decreased in the Control (from 36% to 7%) and T-PL (from 14% to 8%) samples but tended to increase in the T-PP samples (up to 49%) towards the end of the experiment. *Betaproteobacteria* tended to increase (up to 2.6 times more abundant than *Alphaproteobacteria*) towards the post-bloom period and became more abundant than *Alphaproteobacteria* in the Control and T-PL. In T-PP, *Betaproteobacteria* was almost always one order of magnitude less abundant than *Alphaproteobacteria* and up to 8-fold less abundant than that of the samples collected in T-PL. *Actinobacteria* increased towards the post-bloom period, with maximum proportions of 36% and 38% in the Control and T-PL, respectively. *Saccharibacteria* (also known as Candidate division TM7) dominated temporarily in T-PP samples at day 28 (28%), while it was hardly detected in the Control and T-PL.

Among *Alphaproteobacteria*, *Rhizobiales*, *Rhodospirillales*, *Rhodobacterales* and *Sphingomonadales* were the dominant orders (Supplementary Fig. S4). There was no specific shifting pattern of *Rhizobiales* in the Control, T-PL, or T-PP, but it was always more abundant in T-PP than in the Control (up to 35 times) or T-PL (up to 53 times). *Rhodospirillales* decreased, while *Rhodobacterales* increased in T-PL and T-PP.

**Figure 5:**
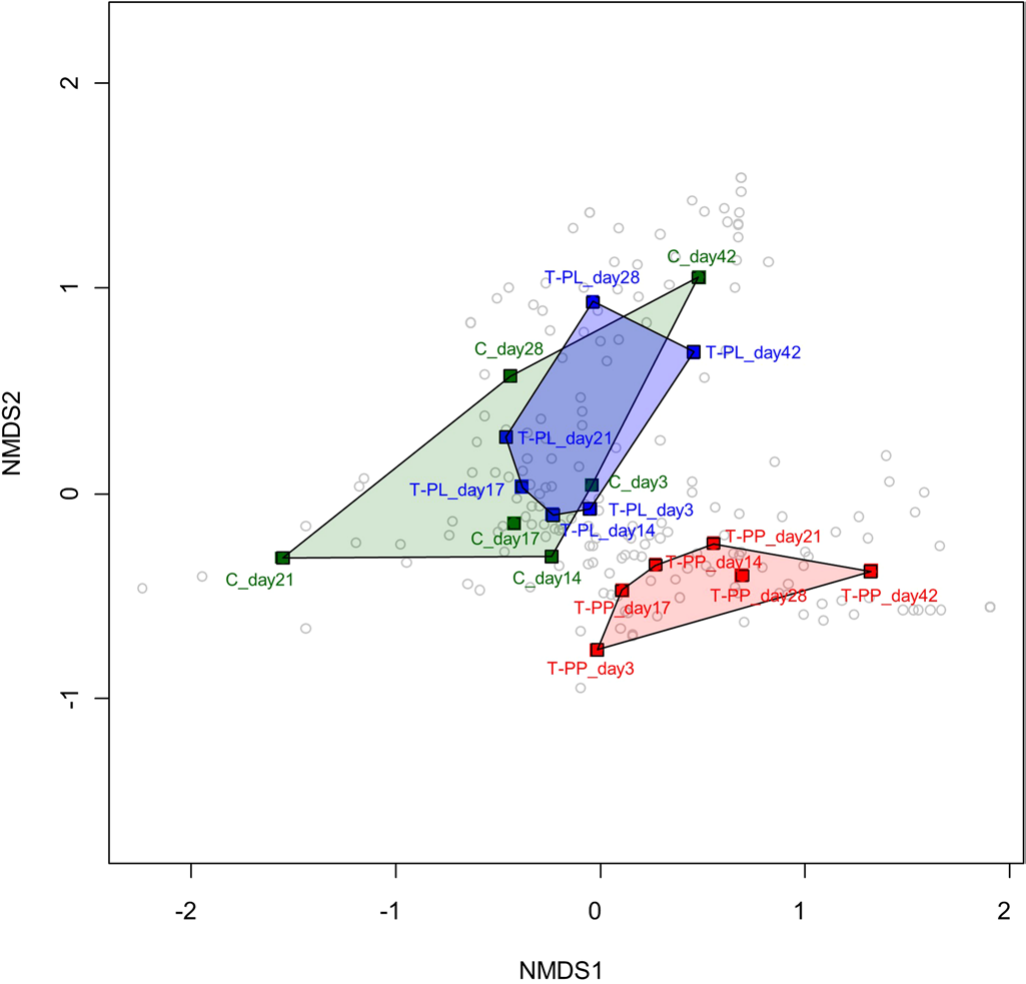
Non-metric multi-dimensional scaling (NMDS) ordinations of the bacterial 16 S rRNA gene community in the Control, T-PL and T-PP samples. The top 100 most abundant OTUs across all samples were used to construct the NMDS. Gray open circles: OTUs. Green, blue, red letters and polygons: Control (﻿C), T-PL and T-PP samples, respectively. Goodness-of-fit of the NMDS was measured using a 2-dimensional stress of 0.083.

NMDS 2-dimensional plots of bacterial communities, based on a metric of dissimilarity among the Control, T-PL and T-PP, revealed the distinct bacterial assemblage in T-PP samples compared to those of Control and T-PL (Fig. 5). Bacterial compositions in Control and T-PL samples showed somewhat similar trajectories during the experiment, although they varied greatly when a huge cyanobacterial bloom developed in Control (day 21).

### *Cyanobacteria* dynamics

**Figure 6:**
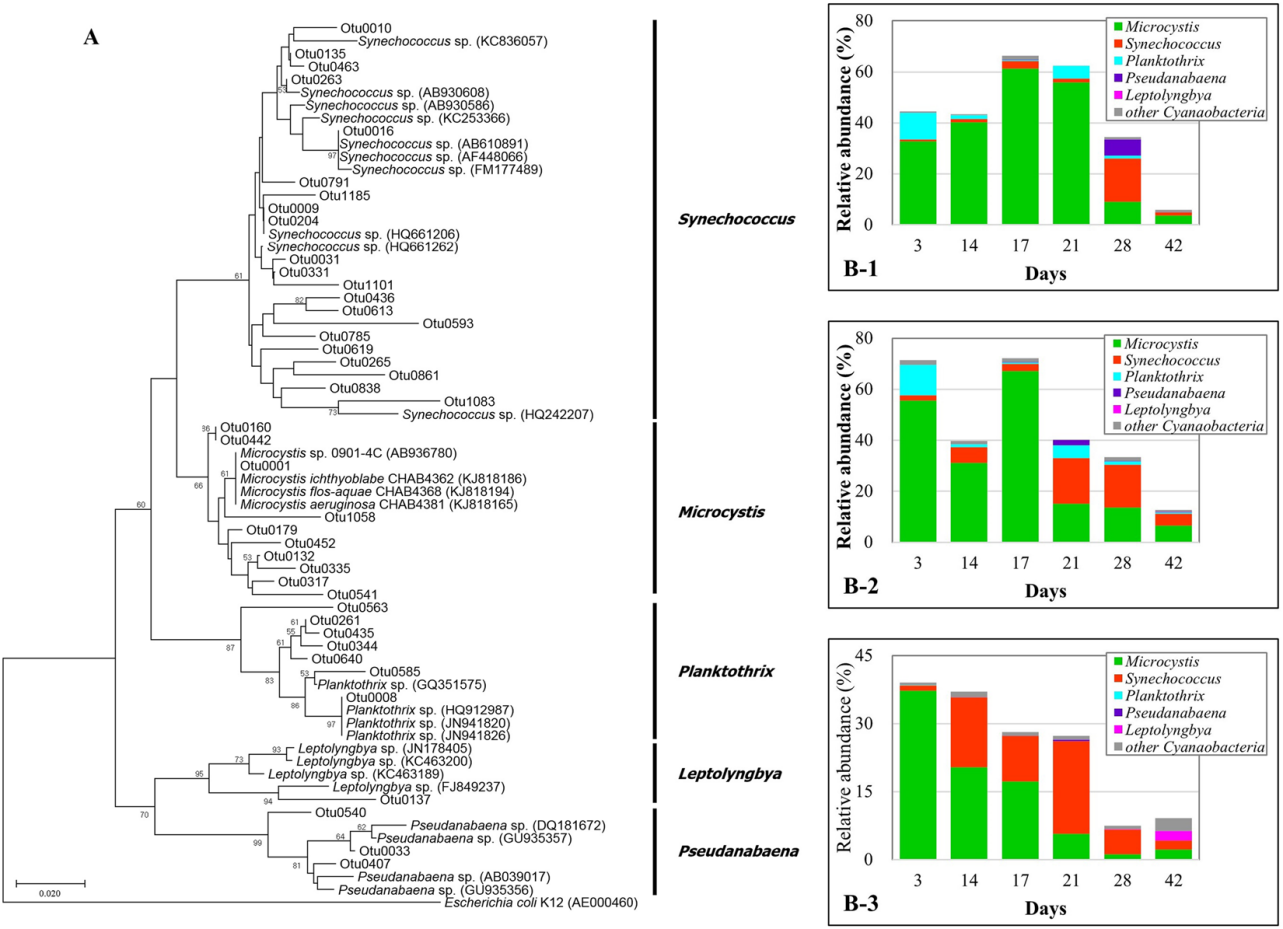
16S rRNA phylogenetic tree of *Cyanobacteria* phylum and the proportions of each assigned genus in the total bacterial communities. Neighbor-joining 16S rRNA phylogenetic tree (**A**) of *Cyanobacteria* phylum. OTUs (relative abundance more than 0.1%) were separated into five different genera: *Microcystis*, *Synechococcus*, *Planktothrix*, *Pseudanabaena* and *Leptolyngbya*. Bootstrap values (expressed as percentages of 1000 replications) > 50% are shown at the branch point. Bar, 0.02 substitutions per nucleotide position. The proportions of genera mentioned above in the Control (B-1), T-PL (B-2) and T-PP (B-3) samples are listed in this figure.

The phylogenetic tree of *Cyanobacteria* constructed from the OTUs with the relative abundance more than 0.1% yielded five different groups: *Microcystis, Synechococcus, Planktothrix, Pseudanabaena* and *Leptolyngbya* (Fig. 6A). Among these five genera, *Microcystis* was predominant, especially in pre-bloom and bloom period samples (ca. 30–70% of total bacteria) (Fig. 6 B-1 to B-3). Its abundance decreased while *Synechococcus* abundance increased towards the post-bloom period in the Control and T-PL. The *Microcystis* proportion constantly decreased and *Synechococcus* followed no specific patterns during the experiment in T-PP. *Planktothrix*, the second dominant *Cyanobacteria* genus in the early experiment phase in the Control and T-PL, tended to decrease towards the post-bloom period, while it was not detected in T-PP samples during the experiment.

### Statistical analysis between bacterial compositions and biophysicochemical factors

**Figure 7:**
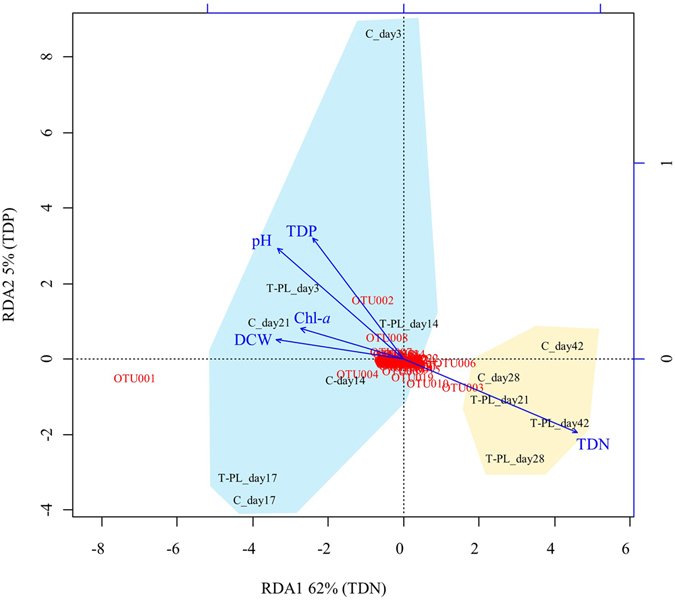
Ordination plot of the redundancy analysis (RDA). The RDA was constructed using relative abundance of 16S rRNA genes and selected environmental factors in the control and treatment planktonic samples. The factors explaining the highest proportion of variability are shown in parentheses in the RDA axes. One cluster (shaded with blue) is comprised of the samples collected from the pre-bloom/bloom periods, while the other cluster (shaded with yellow) is comprised of samples collected from the post-bloom periods.

To evaluate the effects of periphyton on biophysicochemical factors, and finally, on structural changes in the planktonic bacterial community, we performed a redundancy analysis ﻿(RDA) with water samples (Control and T-PL). Based on the RDA model, the selected five environmental factors could be ranked according to their ability to explain variations in the 16S rRNA gene-based bacterial community data (Fig. 7 and Table S1). Together, the first two RDA axes represent 67% of the total variability in the bacterial community structures. The greatest explanatory variable for axis RDA1 was the TDN concentration, while for axis RDA2, it was the TDP concentration. An ANOVA test showed that axis RDA1, TDN concentration and pH were statistically significant in the RDA model (*P* < 0.05), while axis RDA2 and other variables were not (*P* > 0.1). The RDA model, especially axis RDA1, separated 12 samples into two main clusters: pre-bloom/bloom samples (C_day3, C_day14, C_day17, C_day21, T-PL_day3, T-PL_day14, T-PL_day17) and post-bloom samples (C_day28, C-day42, T-PL_day21, T-PL_day28, T-PL_day42). The scores of the individual OTUs indicated that axis RDA1 was mainly represented by the relative abundance of OTU001, OTU003 and OTU006. The relative abundance of OTU001 was higher in pre-bloom/bloom samples and lower in post-bloom samples, while OTU003 and OTU006 were the opposite.

### Correlation analysis of biophysicochemical factors and bacterial groups

A correlation analysis was performed to evaluate the relationship of biophysicochemical factors and bacterial groups (Supplementary Fig. S5). The microcystin concentration was correlated with the relative abundance of *Microcystis* in the Control (*r* = 0.98, *P* < 0.001), while microcystin concentrations in T-PL were not correlated with the relative abundance of *Microcystis* either in T-PL (*r* = 0.53, *P* > 0.1) or in T-PP (*r* = 0.58, *P* > 0.1). The relative abundance of *Cyanobacteria* in T-PL was correlated with the Chl-*a* concentration (*r* = 0.90, *P* < 0.05) and DCW (*r* = 0.93, *P < *0.01) in T-PL. The relative abundance of *Actinobacteria* was negatively correlated with the relative abundance of *Cyanobacteria* (*r* = −0.86, *P* < 0.05) and positively correlated with the TDN concentration (*r* = 0.94, *P* < 0.01) in the Control. The relative abundance of *Cyanobacteria* negatively correlated with the TDN concentration (*r* = −0.85, *P < *0.05) in the Control. The relative abundance of *Actinobacteria* was negatively correlated with *Cyanobacteria* in T-PL (*r* = −0.89, *P* < 0.05).

To evaluate whether bacterial groups and biophysicochemical factors in the water samples have meaningful relationships, we combined data collected from the Control and T-PL (T-PP samples were not used because they are not water samples) (Supplementary Fig. S5J to L) and performed a correlation analysis. The relative abundance of *Actinobacteria* was negatively correlated with *Cyanobacteria* (*r* = −0.84, *P* < 0.001) and positively correlated with the TDN concentration (*r* = 0.86, *P* < 0.001). The relative abundance of *Cyanobacteria* was negatively correlated with the TDN concentration (*r* = −0.75, *P < *0.01).

## Discussion

**Figure 8:**
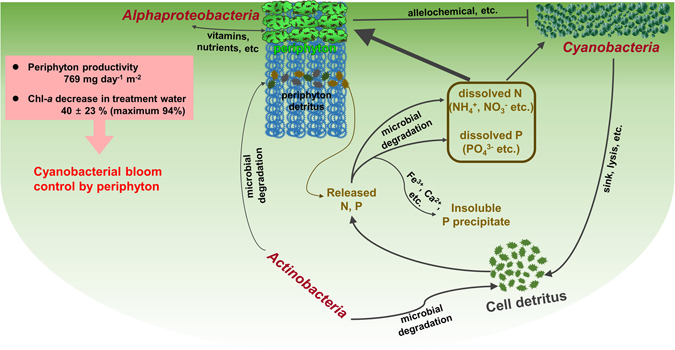
Periphyton effects on harmful cyanobacterial blooms and bacterial assemblages in mesocosms. *Cyanobacteria* detritus that sank to the bottom of the mesocosms will be degraded by *Actinobacteria*. This process will release organic N and P sources into the water. The released N will be further degraded by microbes (e.g., nitrifying bacteria) to several forms of inorganic nitrogen (NH_4_^+^, NO_3_^−^, etc.), resulting in increased TDN in the water. However, some of the released inorganic phosphate will bind to cations such as Fe^3+^ and Ca^2+^ and form insoluble precipitates. These dissolved N and P sources will be used by periphyton, bacteria and other organisms in the mesocosms. When periphyton grows, they would uptake a substantial amount of N and P, which otherwise could be primarily used for cyanobacterial growth. Periphyton prosperity will also harbor their beneficial microbes (e.g., *Alphaproteobacteria*), to obtain essential resources (e.g., vitamins) with an exchange of photosynthates (e.g., carbon sources). Periphyton and their symbiotic microbes decreased Chl-*a* concentrations up to 94% in the treatment water compared to the control water.

A cyanobacterial bloom did not appear in the treatment, while a huge bloom developed in the control, with a maximum Chl-*a* concentration of 2.44 mg l^−1^ (Fig. 2). The Chl-*a* concentration in treatment water (T-PL) decreased approximately 3-fold at the end of the experiment. This decrease accompanied the exponential growth of periphyton flocks in the HBC (Fig. 3, Supplementary Fig. S3). The installed substrates provided a good environment for periphyton attachment and growth. The periphyton also developed its own unique bacterial community structures (Fig. 4, Fig. 5 and Supplementary Fig. S4), including beneficial bacterial groups, to develop symbiosis for nutrient, vitamin and oxygen exchanges. Previous studies have been performed to evaluate the indirect control of cyanobacterial blooms by periphyton growth^17, 23^. Therefore, we assumed that cyanobacterial blooms could be indirectly controlled by the interspecific competition mechanisms, where planktonic organisms compete for the same resources (e.g., Fe(II) availability^25, 26^) and/or through allelochemicals for cyanobacteria (e.g., gramine^27–29^, neo-przewaquinone A^30^, indole^23^, 3-oxo-α-ionone^23^, etc.) (Fig. 8).

Zooplankton was observed rarely in this study. The previous study also revealed that the consumption of cyanobacteria by zooplankton was negligible during periphyton experiments in both batch and field studies^23^. Therefore, we assumed that the changes of cyanobacterial population caused by predators were limited in this study.

The TDP concentration remained relatively constant (Control: 0.16 ± 0.01 mg l^−1^, T-PL: 0.15 ± 0.01 mg l^−1^) (Fig. 1) and always exceeded the hypereutrophic criteria (100 > μg l^−1^)^31^. Therefore, we assumed that phosphate was not a limiting factor for microalgae in this study. The continuous increase of the TDN concentration (approximately 1.6 times) was observed after the cyanobacterial bloom collapsed in both mesocosms. The relative abundance of *Cyanobacteria* was negatively correlated with TDN concentrations in the Control (Supplementary Fig. S5G), and decreased when TDN concentrations increased in T-PL (Supplementary Fig. S5I). Since the mesocosms used in this study were closed systems, the inflow of N sources from outside the mesocosm was regarded as negligible. This increase in TDN concentrations was most likely caused by microbial decomposition of microalgae debris (mainly *Cyanobacteria*), which might sink into the bottom of mesocosms because of the loss of their buoyancy (Fig. 8). This process releases inorganic N compounds such as NH_4_^+^ and NO_3_^−^^32, 33^ as dissolved nitrogen sources, which can be readily assimilated for algal growth. Phosphate, the end product of decomposition of P compounds, might form insoluble complexes with Fe^3+^ and/or Ca^2+^ under oxic conditions, thereby reducing the amount of bioavailable P sources.

Planktonic bacterial communities in pre-bloom/bloom periods differed from the post-bloom period and they were mainly influenced by TDN concentrations (Fig. 7). Our results were consistent with the previous observations that dissolved nitrogen concentrations, combined with other biogeochemical factors, were responsible for the bacterial community shifts in freshwaters^34, 35^. Apparently, the increased TDN concentrations which might be caused by microbial decomposition of cyanobacterial debris drove the shifts of bacterial communities, such as decomposers (e.g., *Actinobacteria*, to be discussed later) in water samples (Fig. 1 and Fig. 4).

*Actinobacteria*, one of the typical freshwater bacterial groups, made up an average of 9 ± 14% and 14 ± 14% of the total bacteria in the Control and T-PL samples, respectively. The relative abundance of *Actinobacteria* was negatively correlated with *Cyanobacteria* both in the Control and T-PL (Supplementary Fig. S5E and H). Additionally, the relative abundance of *Actinobacteria* in T-PL apparently increased when *Cyanobacteria* decreased in T-PP. It has been reported that *Actinobacteria* possess specific genes involved in the uptake and metabolism of N-rich organic compounds^36^. *Cyanobacteria* detritus could be a good energy source for *Actinobacteria* (and/or other bacteria) growth, and their biodegradation byproducts, released into the water as organic/inorganic nitrogenous compounds, seem to further increase TDN concentrations in the water (Fig. 8).

Microscopic observations revealed that cyanoba﻿cteria, especially *Microcystis*, were the predominant microalgae and the major contributor to Chl-*a* concentrations in both Control and T-PL (Supplementary Fig. S2A, Fig. 6 B-1 and B-2). Water samples in the Control had an average of 3.33 ± 5.39 times higher Chl-*a* concentrations than those in T-PL samples (Fig. 2A). These data suggested that the water in the control contained more *Microcystis* than the treatment water.

*Cyanobacteria* (or *Microcystis*) maintained similar proportions at the phylum level, even when the Chl-*a* peak appeared in the Control (day 21) (Fig. 4A, Fig. 6 B-1). However, the NMDS, which was constructed with the top 100 most abundant OTUs, showed that the bacterial community structure was considerably different at day 21 compared with other samples (Fig. 5). *Cyanobacteria*-associated bacteria (e.g., Rhizobacteria, *Roseomonas*) increased with *Cyanobacteria*, and this increase further affected the overall bacterial community structures. However, these changes could not be represented at the phylum level, but only at the OTU level.

We found that the relative abundance of *Rhodobacterales* apparently increased with Chl-*a* concentrations in T-PP, although there was no significant correlation, most likely due to the small sample size (Supplementary Fig. S5M). Among *Rhodobacterales*, 70–90% OTUs were assigned to *Rhodobacter* and *Tabrizicola*. Previous studies reported that *Rhodobacter* spp. could synthesize vitamins^37^, and *Tabrizicola* spp. could grow without any additional vitamin supplements^38^. Our results, combined with previous findings, indicated that vitamins (or other metabolites) synthesized by periphyton-associated *Rhodobacterales* could be supplied for their symbionts (diatoms, *Scenedesmus* spp. and *Oedogonium* spp., etc.) with an exchange of phytoplankton-derived photosynthates (e.g., carbon sources) to accomplish mutualism and symbiosis (Fig. 8).

We obtained the following key results in this study. First, periphytic bacterial populations were quite different from the planktonic bacterial populations. *Alphaproteobacteria* always dominated and some unique bacterial groups (e.g., *Saccharibacteria*) temporarily dominated periphytic bacteria. Second, planktonic bacteria compositions in the experimental mesocosms varied considerably during the pre-bloom/bloom/post-bloom periods. Generally, *Cyanobacteria* and *Alphaproteobacteria*, the dominant groups during the pre-bloom and bloom periods, decreased, while *Actinobacteria* increased towards the post-bloom period. Third, planktonic bacterial compositions were mainly influenced by TDN concentrations which could be derived from microbial decomposition of cyanobacterial debris. This is the first report of the bacterial roles in the control of cyanobacterial blooms using periphyton on the field scale. The possible mechanisms of bloom control by periphyton and their symbiotic bacterial partners were also discussed. In conclusion, periphyton formation could create a unique microbial community that interacts with the planktonic microbial community, thereby contributing to microalgal species diversity, ecosystem health and finally bloom control.

## Methods

### Experimental site and mesocosm setup

Two mesocosms were installed in Lake Seo (37°16′36″N, 126°59′27″E), Suwon, Gyeonggi Province, South Korea, on 21 July, 2014. The surface area of Lake Seo is 0.19 km^2^, and the average depth is 1.72 m. For the last 5 years, its average concentrations of total nitrogen, total phosphorus and Chlorophyll-*a* (Chl-*a*) were 6.7 mg l^−1^, 0.21 mg l^−1^ and 68.3 μg l^−1^ (87.2 μg l^−1^ in the summer from June to September), respectively (data obtained from Water Information System, http://water.nier.go.kr), satisfying the hypereutrophic criteria of the Organization for Economic Co-operation and Development (OECD)^31^. Each mesocosm (5 m × 10 m × 1.5 m (depth)) was constructed with an oil fence made of polyvinyl chloride. The bottom of the mesocosm was also sealed with an oil fence, completely separating the water inside the mesocosm from the outside lake water. Both the control and treatment mesocosms were filled with approximately 75 tons of lake water. The treatment mesocosm was equipped with a hanging bio-contactor (HBC, HanilEST, Korea), originally used for biofilm formation in wastewater treatment, to induce periphyton attachment and growth. The HBC is made of polyvinylidene chloride fiber and has a hairy structure to maximize the surface area for attachment. A total of 200 HBCs with a length of 2 m were installed vertically in 10 rows with 20 HBCs in each row inside the treatment mesocosm (Fig. S1). The periphyton was not deployed artificially but indigenous microalgae and bacteria in Lake Seo attached and colonized the HBCs to form a natural periphyton.

### Sampling and monitoring of water quality

Water and periphyton samples were collected every 3 to 4 days for 42 days from 24 June to 1 September, 2014. Water samples were collected from the surface about a depth of 20 cm after some mixing at three different places in the mesocosm. Since the heterogeneous distribution of floating scums could cause some biases in the measurements, the surface water was stirred gently 3 times in different directions, using a 4-L sampling bottle in an area of 1 m × 1 m, just before sampling. Water samples from the control, treatment water and treatment periphyton were designated Control, T-PL and T-PP, respectively. The water temperature and pH were measured with a portable instrument (Multi 3410, WTW GmbH, Germany). The water samples for physicochemical analysis were collected from the surface water within a 20-cm depth using 4-L water collection bottles and stored at 4 °C until analysis. The water samples were filtered through a 0.45-μm syringe filter, and the filtrate was used for the measurement of total dissolved nitrogen (TDN) and total dissolved phosphorus (TDP). The TDN concentration was analyzed using a multi N/C^®^ 3100 (Analytik, Jena, Germany). The TDP concentration was measured after persulfate oxidation into orthophosphate, using the phosphomolybdate method^37, 39^. The Chl-*a* concentration was measured using a fluorometer (Turner 450, Barnstead/Thermolyne, Dubuque, IA) after filtration through a GF/C filter (Whatman, UK) and extraction using a chloroform-methanol mixture (2:1, v/v)^40^.

For the periphyton analysis, three 30-cm strips from the water surface were cut off from separate HBC strings at different positions and transported to the laboratory in sterilized plastic bags at 4 °C. The periphyton was stripped from the HBC pieces in a stomacher bag containing 150 mL of sterile water using a BagMixer® 400 (Interscience Co., Saint Nom, France) and homogenized for 20 s^41^. The algal diversity and abundance were estimated under a microscope (Nikon Corp., Tokyo, Japan). The Chl-*a* and dry cell weight (DCW) of the periphyton were also measured.

The concentration of extracellular microcystin was measured using an enzyme-linked immunosorbent assay (ELISA; EnviroLogix Inc., USA) after filtration of the water samples through a GF/C filter. The ELISA plate was read at 450 nm using a microplate absorbance reader (Sunrise, Tecan, Switzerland)^42^.

### DNA extraction

Genomic DNA was extracted using a FastDNA™ SPIN Kit for Soil DNA extraction (MP Biomedicals, USA) according to the manufacturer’s instructions. Membrane filters that were used to collect microbes were cut into pieces with sterile surgical blades. The membrane pieces were then put into the tubes supplied by the kit, which contained microbeads to smash membrane pieces to efficiently extract DNA.

### Bacterial 16S rRNA gene analysis

16S rRNA gene was targeted to determine the compositions of the bacterial community. Bacterial 16S rRNA genes were amplified for 25 cycles using the barcoded primer set 27F: 5′-AGAGTTTGATCMTGGCTCAG-3′ and the reverse primer 519R: 5′-GWATTACCGCGGCKGCTG-3′^43^. This primer set targets the V1-V3 region of the bacterial 16S rRNA gene. Each sample was marked with a specific barcode so that it could be recognized after pyrosequencing. The PCR products were purified using the Agencourt AMPure XP (Beckman Coulter, USA) according to the manufacturer’s instructions. The prepared DNA libraries were sequenced using 454 GS Junior (Roche Applied Science, USA) according to the manufacturer’s instructions.

The 16S rRNA gene sequences obtained from pyrosequencing were processed using mothur v.1.36.1^44^ following the standard operating procedure (SOP) proposed by Schloss, *et al*.^45^. Low-quality sequences were removed from the bacterial community analysis if they had a read quality score under 30, or contained ambiguous characters, or contained more than two mismatches to the forward primer, or one mismatch to the barcode, or were under 300 bp or over 550 bp. The pre-cluster method^46^ was used to reduce the sequencing noise produced by pyrosequencing. The chimera.uchime^47^ was used to identify and remove chimera sequences. The average read length was approximately 300 bp after the barcode and primer sequences were trimmed. The SILVA database (silva.nr_v123) was used to align and classify the obtained sequences. The similarity cutoff of > 97% was used to assign the same OTUs. Within the generated OTU table, about 30% of OTUs of total bacteria were assigned as “unclassified” forms under *Cyanobacteria* phylum and/or *Bacteria* kingdom. These OTU sequences were manually searched against NCBI database to obtain the accession numbers of the most closely related sequences. The obtained NCBI accession numbers were again searched against SILVA database (https://www.arb-silva.de) to double-check their taxonomic positions. If the sequences were assigned to Chloroplast or Mitochondria, they were excluded in our study.

A considerable proportion of the OTUs classified as *Cyanobacteria* was under the unclassified position; therefore, we collected sequences under the *Cyanobacteria* phylum with relative abundances > 0.5% to construct a phylogenetic tree using the neighbor-joining method for further analysis.

### Nucleotide sequence accession numbers

Bacterial 16S rRNA gene sequences and accompanying metadata have been deposited in the DDBJ (http://www.ddbj.nig.ac.jp/) Sequence Read Archive under project number DRA005310.

### Statistical analysis

To compare discrepancies in the bacterial communities in obtained samples, non-metric multidimensional scaling (NMDS) was performed using R’s Vegan package^48^. The top 100 most abundant OTUs were used to calculate Bray-Curtis dissimilarities. To investigate the potential environmental drivers of bacterial community compositions in water samples (Control and T-PL), a redundancy analysis (RDA) was performed using R’s BiodiversityR package^49, 50^. Bacterial 16S rRNA gene data and the collected environmental factors were used to perform an RDA analysis. The environmental factors were standardized by subtracting the mean value and then dividing by the standard deviation of the variable. To evaluate differences in the bacterial communities among Control, T-PL and T-PP, we used the Bray-Curtis dissimilarity distance matrix based on the relative abundance of 16S rRNA data to construct the dendrogram. The dendrogram was constructed using the UPGMA algorithm. Pearson correlation coefficient (*r*) and significance (*P*) values were calculated using R’s Hmisc package^51^ and the PerformanceAnalytics packages^52^.

## Electronic supplementary material

Supplementary Information 
