# Supplementary material for: Periphyton effects on bacterial assemblages and harmful cyanobacterial blooms in a eutrophic freshwater lake: a mesocosm study
Source: Sci Rep. 2017 Aug 10;7:7827. doi: 10.1038/s41598-017-08083-x (PMC5552679; doi:10.1038/s41598-017-08083-x)
Supplement: Supplementary file 1 — Supplementary Information [file 41598_2017_8083_MOESM1_ESM.doc]

**Periphyton effects on bacterial assemblages and harmful cyanobacterial blooms in a eutrophic freshwater lake: a mesocosm study**

Yingshun Cui, Long Jin, So-Ra Ko, Seong-Jun Chun, Hyung-Seok Oh, Chang Soo Lee, Ankita Srivastava, Hee-Mock Oh & Chi-Yong Ahn

**Supplementary information**


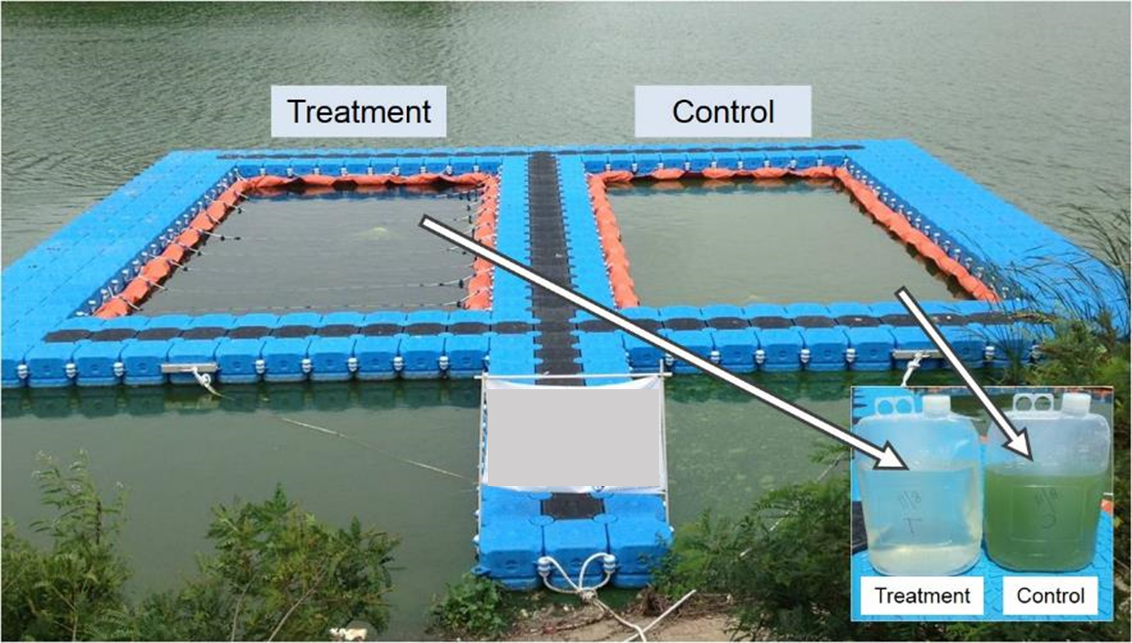


#### Fig. S1. Differences in cyanobacterial growth in the surface water between the treatment (periphyton) and control mesocosms on day 21.


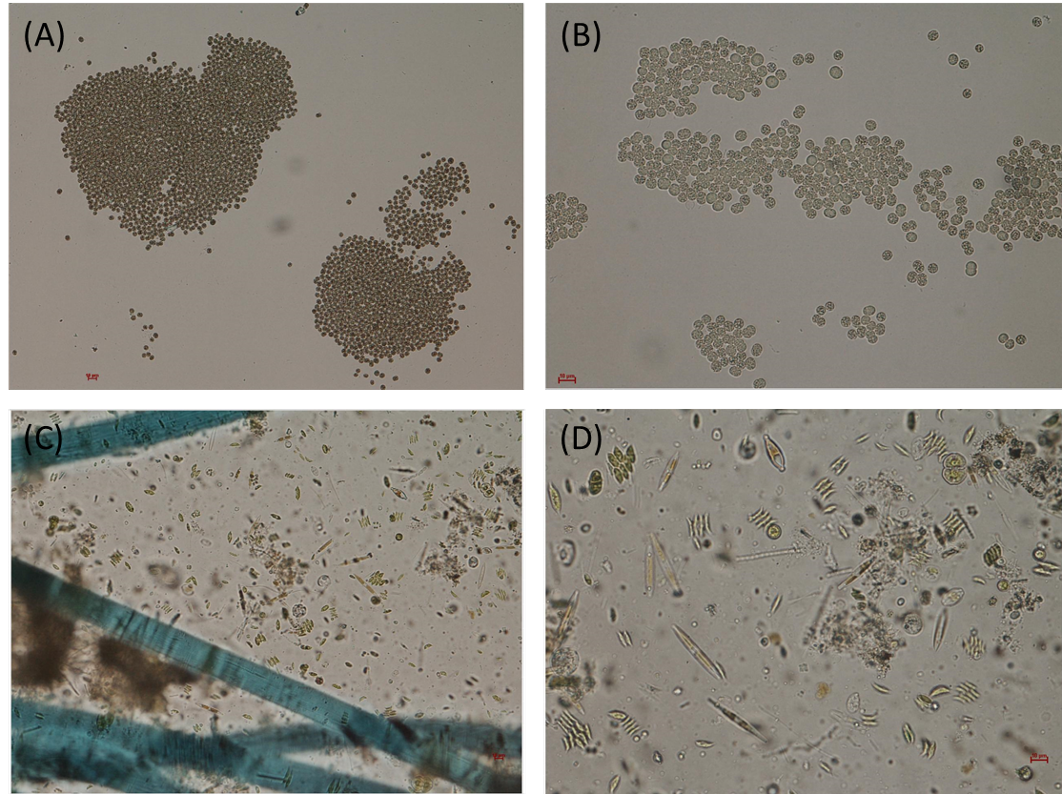


#### Fig. S2. Differences in microalgae diversity between the surface water of the control ((A) and (B)) and periphyton of the treatment ((C) and (D)) mesocosm on day 21. The dominant microalgae in the surface water of treatment mesocosm also comprised of cyanobacteria (similar to the control), therefore, the microscopic pictures of these samples were not shown here.


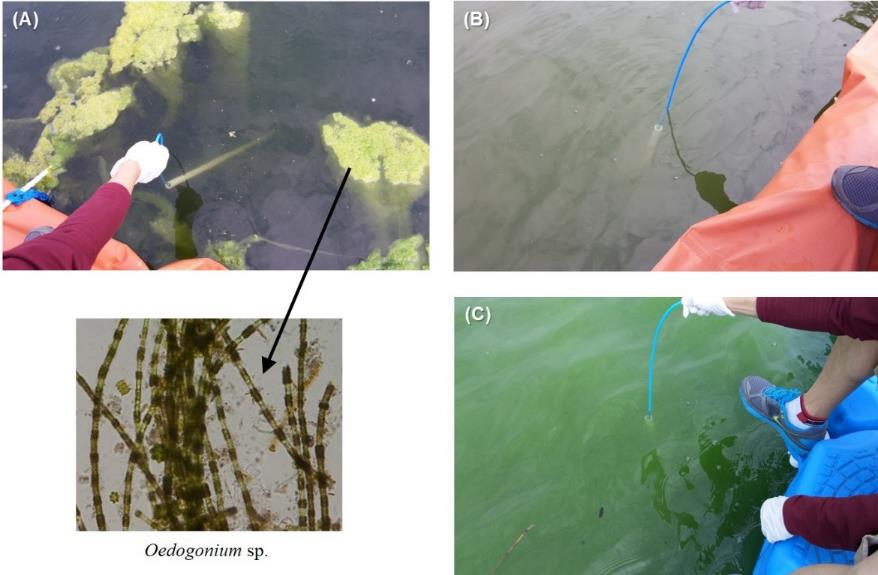


#### Fig. S3. Comparison of transparency in the surface water between (A) treatment, (B) control and (C) lake water outside the mesocosm. Microscopic image of the filamentous algae from the treatment mesocosm is also shown

####

#### Fig. S4. Bacterial community composition of each sample. Orders are listed in this figure. Cyanobacteria were separated into *Microcystis*, *Synechococcus*, *Planktothrix*, *Pseudanabaena* and *Leptolyngbya* according to the phylogenetic tree. a) Control, b) T-PL and c) T-PP samples.

#### Fig. S5. Correlation analysis of the relative abundances of bacterial groups and environmental factors.

#### Table S1. Summary of RDA analysis and the obtained scores.

| **Partitioning of variance:** | |  |  |  |  |  |
| --- | --- | --- | --- | --- | --- | --- |
|  | Inertia | Proportion |  |  |  |  |
| Total | 788.1 | 1 |  |  |  |  |
| Constrained | 583.4 | 0.7403 |  |  |  |  |
| Unconstrained | 204.7 | 0.2597 |  |  |  |  |
|  |  |  |  |  |  |  |
| **Eigenvalues, and their contribution to the variance** | | | |  |  |  |
|  |  |  |  |  |  |  |
| **Importance of components:** | |  |  |  |  |  |
|  | RDA1 | RDA2 | RDA3 | RDA4 | RDA5 |  |
| Eigenvalue | 491.9293 | 40.8238 | 36.0571 | 10.1697 | 4.46023 |  |
| Proportion Explained | 0.6242 | 0.0518 | 0.04575 | 0.0129 | 0.00566 |  |
| Cumulative Proportion | 0.6242 | 0.676 | 0.72175 | 0.7347 | 0.74032 |  |
|  |  |  |  |  |  |  |
|  | PC1 | PC2 | PC3 | PC4 | PC5 | PC6 |
| Eigenvalue | 133.5798 | 36.47257 | 18.30568 | 9.9291 | 4.8895 | 1.47751 |
| Proportion Explained | 0.1695 | 0.04628 | 0.02323 | 0.0126 | 0.0062 | 0.00187 |
| Cumulative Proportion | 0.9098 | 0.95609 | 0.97932 | 0.9919 | 0.9981 | 1 |
|  |  |  |  |  |  |  |
|  |  |  |  |  |  |  |
|  |  |  |  |  |  |  |
|  |  |  |  |  |  |  |
|  |  |  |  |  |  |  |
| **Accumulated constrained eigenvalues** | | |  |  |  |  |
| **Importance of components:** | |  |  |  |  |  |
|  | RDA1 | RDA2 | RDA3 | RDA4 | RDA5 |  |
| Eigenvalue | 491.9293 | 40.8238 | 36.0571 | 10.16975 | 4.46023 |  |
| Proportion Explained | 0.8431 | 0.06997 | 0.0618 | 0.01743 | 0.00764 |  |
| Cumulative Proportion | 0.8431 | 0.91312 | 0.9749 | 0.99236 | 1 |  |
|  |  |  |  |  |  |  |
|  |  |  |  |  |  |  |
| **Species scores** |  |  |  |  |  |  |
|  |  |  |  |  |  |  |
|  | RDA1 | RDA2 | RDA3 | RDA4 | RDA5 |  |
| OTU001 | -8.86E+00 | -1.87E+00 | 1.87E+00 | 1.12E+00 | -7.70E-01 |  |
| OTU002 | -8.75E-01 | 7.15E+00 | -2.77E-01 | 2.58E+00 | 2.46E+00 |  |
| OTU008 | -3.93E-01 | 2.86E+00 | 2.41E-01 | -3.72E-01 | 1.36E-01 |  |
| OTU014 | 1.71E-01 | 9.71E-01 | 1.11E+00 | 7.31E-02 | 1.74E+00 |  |
| OTU018 | -3.26E-02 | 9.59E-01 | -3.61E-01 | -4.66E-01 | -1.23E+00 |  |
| OTU007 | -2.64E-01 | 1.11E+00 | -6.26E-01 | -4.12E+00 | 9.21E-01 |  |
| OTU037 | -2.10E-02 | 3.49E-01 | -2.28E-01 | 1.15E-01 | -1.25E+00 |  |
| OTU048 | 4.99E-03 | 3.33E-01 | 1.77E-02 | -2.20E-02 | -4.65E-01 |  |
| OTU015 | 6.83E-01 | -8.07E-01 | 6.96E-01 | -3.47E-01 | 7.55E-01 |  |
| OTU005 | -2.88E-02 | 5.31E-01 | -3.47E-01 | -6.51E-01 | -5.85E-01 |  |
| OTU021 | -3.86E-02 | 6.38E-01 | -3.38E-01 | -5.87E-01 | -7.05E-01 |  |
| OTU081 | -1.25E-02 | 2.66E-01 | -2.55E-02 | 3.10E-02 | -3.79E-01 |  |
| OTU003 | 2.13E+00 | -2.92E+00 | 4.17E+00 | -1.67E+00 | 1.59E+00 |  |
| OTU012 | -1.60E-01 | 9.80E-01 | -1.04E+00 | -4.10E+00 | -3.17E-01 |  |
| OTU027 | -5.16E-03 | 2.27E-01 | -3.64E-02 | 3.77E-02 | -2.53E-01 |  |
| OTU045 | -2.42E-02 | 2.50E-01 | -1.64E-02 | -2.72E-01 | -2.80E-01 |  |
| OTU020 | 5.08E-01 | -5.71E-01 | 4.85E-01 | 5.45E-02 | 2.77E-01 |  |
| OTU010 | 9.39E-01 | -2.52E+00 | -4.16E+00 | 2.43E+00 | 1.17E+00 |  |
| OTU013 | -6.87E-02 | 7.43E-02 | 5.88E-02 | -2.25E+00 | -4.44E-01 |  |
| OTU023 | 2.77E-01 | 1.68E-01 | 7.52E-01 | 5.09E-01 | -4.23E-01 |  |
| OTU042 | -4.51E-02 | 9.66E-02 | -2.95E-02 | -4.74E-01 | -4.26E-01 |  |
| OTU069 | 4.73E-04 | 1.02E-01 | 1.85E-02 | 7.32E-02 | -2.84E-01 |  |
| OTU044 | -5.90E-02 | 2.98E-01 | -3.38E-01 | -3.63E-01 | -7.04E-01 |  |
| OTU049 | -1.77E-03 | -6.08E-02 | -3.95E-02 | -6.11E-02 | -9.20E-01 |  |
| OTU039 | -7.79E-02 | 1.07E-01 | -2.63E-01 | -8.36E-01 | -2.07E-01 |  |
| OTU025 | -1.43E-01 | 1.81E-01 | -3.94E-01 | -1.12E+00 | -9.91E-01 |  |
| OTU026 | -3.44E-02 | 1.66E-01 | -1.13E-01 | -7.51E-01 | 1.10E-01 |  |
| OTU034 | -4.74E-02 | 5.07E-02 | -8.23E-02 | -6.76E-01 | 7.70E-02 |  |
| OTU006 | 1.86E+00 | -1.56E-01 | 4.67E+00 | 2.76E+00 | -3.81E-01 |  |
| OTU019 | 4.47E-01 | -1.82E+00 | -3.52E+00 | 2.59E+00 | 1.33E+00 |  |
| OTU041 | 1.11E-01 | -1.40E-01 | -2.46E-01 | 4.51E-01 | -7.73E-02 |  |
| OTU078 | -1.72E-02 | 8.22E-02 | -4.46E-02 | -5.54E-01 | 2.01E-01 |  |
| OTU053 | 2.37E-02 | -1.23E-01 | 1.67E-01 | 3.62E-01 | -3.65E-01 |  |
| OTU056 | -2.16E-02 | 2.36E-01 | -1.90E-01 | -4.78E-01 | -5.78E-02 |  |
| OTU057 | 1.70E-03 | 3.22E-02 | -1.24E-01 | -4.21E-01 | -1.77E-02 |  |
| OTU077 | 6.67E-02 | -1.17E-02 | -7.05E-02 | 2.71E-01 | -7.50E-02 |  |
| OTU142 | -8.58E-03 | 6.77E-02 | -2.34E-02 | 4.46E-03 | -1.42E-01 |  |
| OTU072 | -8.71E-03 | 4.50E-02 | 5.41E-03 | -1.27E-01 | -6.58E-02 |  |
| OTU153 | -2.48E-03 | 4.30E-02 | 4.17E-03 | -1.73E-02 | -5.39E-02 |  |
| OTU009 | 1.38E-01 | -1.06E+00 | -1.14E+00 | 6.77E-01 | -2.36E+00 |  |
| OTU051 | -2.53E-02 | 2.28E-01 | -2.46E-01 | -2.77E-01 | -3.56E-01 |  |
| OTU087 | -2.84E-02 | 1.66E-02 | 3.94E-02 | 3.11E-01 | 4.20E-01 |  |
| OTU090 | 7.10E-02 | -8.54E-02 | -7.85E-02 | 7.64E-02 | 1.43E-01 |  |
| OTU170 | 4.19E-03 | 8.31E-03 | 1.40E-02 | -3.03E-02 | -1.90E-01 |  |
| OTU060 | -8.41E-03 | 6.86E-02 | -1.22E-01 | -2.26E-01 | -1.15E-01 |  |
| OTU031 | -8.08E-02 | -2.87E-01 | -2.29E-01 | 8.95E-02 | -9.92E-01 |  |
| OTU032 | 4.10E-01 | -2.27E-01 | -1.01E-01 | 1.33E+00 | 3.81E-01 |  |
| OTU036 | 1.46E-01 | -3.89E-02 | 1.74E-01 | 9.52E-04 | -5.32E-01 |  |
| OTU043 | 2.63E-01 | 1.59E-01 | 6.61E-01 | 7.54E-01 | -2.61E-01 |  |
| OTU064 | -5.84E-02 | -1.48E-01 | -1.94E-02 | 9.15E-02 | -6.02E-01 |  |
| OTU068 | -3.69E-03 | 6.81E-02 | -7.17E-02 | -3.28E-01 | 7.72E-02 |  |
| OTU109 | -1.75E-03 | 2.11E-02 | -1.85E-03 | -2.49E-02 | -1.33E-02 |  |
| OTU141 | 9.53E-03 | 4.09E-02 | 2.04E-02 | 3.87E-02 | -4.53E-02 |  |
| OTU028 | 3.65E-01 | -5.61E-01 | -1.19E-01 | 4.30E-01 | 6.86E-01 |  |
| OTU059 | -5.91E-02 | -1.72E-01 | -2.07E-01 | 6.03E-01 | 1.72E-01 |  |
| OTU070 | -1.63E-02 | -1.34E-01 | 7.90E-02 | 3.68E-01 | 5.00E-02 |  |
| OTU073 | -4.62E-03 | 7.34E-02 | -6.64E-02 | -2.08E-01 | 6.53E-02 |  |
| OTU082 | 9.53E-04 | 3.09E-03 | 2.07E-03 | -1.33E-02 | -1.31E-02 |  |
| OTU083 | -1.98E-02 | -9.29E-03 | -1.52E-02 | -2.78E-01 | -1.45E-01 |  |
| OTU104 | 5.70E-02 | -2.80E-02 | 2.04E-01 | -6.92E-02 | 4.25E-02 |  |
| OTU129 | -4.44E-03 | 1.17E-01 | -9.92E-02 | -1.91E-01 | -5.52E-02 |  |
| OTU226 | 2.89E-02 | -6.31E-02 | -1.48E-01 | 1.15E-01 | 1.15E-01 |  |
| OTU273 | -1.58E-03 | 5.50E-03 | -6.91E-04 | 5.70E-03 | -2.48E-02 |  |
| OTU004 | -1.40E+00 | -1.40E+00 | 7.28E-02 | -1.42E+00 | 6.43E+00 |  |
| OTU016 | 1.56E-02 | -3.84E-02 | -1.12E-02 | -4.30E-02 | 1.58E-03 |  |
| OTU017 | 2.15E-02 | 8.99E-03 | 3.53E-02 | 4.11E-02 | -3.21E-03 |  |
| OTU022 | 6.25E-01 | 4.85E-01 | 1.92E+00 | 1.81E+00 | -7.74E-01 |  |
| OTU024 | 5.28E-01 | 1.82E-01 | 1.77E+00 | 7.89E-01 | -2.93E-01 |  |
| OTU029 | 3.65E-01 | -3.47E-01 | 1.38E+00 | -8.34E-01 | 5.77E-01 |  |
| OTU030 | 6.67E-02 | 4.82E-01 | -5.62E-01 | -2.91E-01 | -1.16E+00 |  |
| OTU033 | 2.88E-02 | -4.91E-01 | -7.21E-01 | 7.35E-01 | -1.24E+00 |  |
| OTU038 | 4.86E-02 | -8.98E-02 | 1.72E-01 | -1.88E-01 | 1.07E-01 |  |
| OTU047 | 2.49E-01 | 3.05E-01 | 7.64E-01 | 1.00E+00 | -4.92E-01 |  |
| OTU050 | 3.54E-02 | -1.37E-01 | -2.59E-01 | 1.91E-01 | 1.15E-01 |  |
| OTU052 | 2.27E-01 | 2.78E-01 | 6.96E-01 | 9.13E-01 | -4.48E-01 |  |
| OTU054 | 1.87E-01 | -2.75E-01 | -7.84E-03 | 1.57E-01 | 4.38E-01 |  |
| OTU058 | 1.83E-02 | -5.85E-02 | 5.84E-02 | -1.16E-01 | 7.22E-02 |  |
| OTU061 | 1.13E-01 | -2.12E-01 | 4.62E-01 | -5.62E-01 | 3.50E-01 |  |
| OTU062 | -8.41E-03 | 3.40E-01 | -3.48E-01 | -4.85E-01 | -3.56E-02 |  |
| OTU063 | 1.19E-02 | 2.97E-02 | -4.35E-03 | -8.67E-02 | 1.36E-02 |  |
| OTU066 | 1.30E-01 | -3.41E-01 | -6.75E-01 | 5.14E-01 | 5.19E-01 |  |
| OTU067 | 1.14E-01 | 5.42E-02 | 3.79E-01 | 2.11E-01 | -8.55E-02 |  |
| OTU071 | 3.55E-02 | 2.35E-02 | 4.76E-02 | -1.55E-01 | 1.66E-01 |  |
| OTU074 | 1.13E-01 | 3.67E-02 | 2.58E-01 | 2.98E-01 | -5.46E-02 |  |
| OTU075 | 1.02E-01 | -2.88E-01 | -4.20E-01 | 2.88E-01 | 3.54E-01 |  |
| OTU076 | -9.53E-04 | -9.88E-02 | -1.98E-01 | 1.40E-01 | -2.70E-01 |  |
| OTU079 | 9.73E-02 | -6.49E-03 | 3.40E-01 | 2.71E-02 | 1.33E-02 |  |
| OTU080 | 1.02E-01 | 5.86E-02 | 3.34E-01 | 2.18E-01 | -9.30E-02 |  |
| OTU084 | 4.13E-05 | -2.54E-03 | -5.50E-03 | 5.00E-03 | -6.15E-03 |  |
| OTU085 | 2.67E-03 | 8.72E-03 | -6.04E-03 | 9.16E-03 | -1.42E-02 |  |
| OTU086 | 1.17E-02 | -9.64E-02 | 3.57E-02 | -1.32E-01 | -4.20E-01 |  |
| OTU088 | -5.61E-02 | 9.27E-02 | -3.71E-02 | 2.44E-01 | 4.99E-01 |  |
| OTU092 | 9.16E-03 | -2.69E-02 | -2.51E-02 | -4.05E-02 | 1.10E-02 |  |
| OTU093 | 6.74E-02 | -1.49E-01 | 2.83E-01 | -4.00E-01 | 2.46E-01 |  |
| OTU094 | 8.30E-02 | 2.43E-03 | 2.87E-01 | 4.62E-02 | -1.68E-03 |  |
| OTU095 | -8.88E-02 | -1.22E-02 | 1.24E-01 | 5.83E-01 | 1.26E+00 |  |
| OTU096 | 4.69E-02 | -1.67E-01 | -1.94E-01 | 1.67E-01 | -2.80E-02 |  |
| OTU097 | -1.21E-02 | -2.79E-02 | -4.39E-02 | -2.14E-01 | -1.62E-01 |  |
| OTU099 | 8.64E-02 | 9.93E-02 | 2.67E-01 | 3.29E-01 | -1.60E-01 |  |
| OTU100 | -4.97E-03 | 3.34E-02 | -4.46E-02 | -2.30E-01 | 8.49E-02 |  |
| OTU101 | 5.83E-03 | -9.58E-02 | -9.93E-02 | 1.19E-01 | -3.04E-01 |  |
| OTU102 | 8.62E-02 | 3.85E-02 | 1.36E-01 | 3.25E-01 | -5.76E-02 |  |
| OTU103 | -1.19E-03 | 9.64E-03 | -1.02E-02 | -4.81E-02 | 2.15E-02 |  |
| OTU106 | -5.02E-04 | -2.63E-02 | 6.67E-03 | -8.40E-03 | -3.47E-01 |  |
| OTU107 | 1.22E-02 | -3.64E-02 | 6.28E-02 | -1.23E-01 | 5.01E-02 |  |
| OTU110 | 6.00E-02 | -1.97E-01 | -4.07E-01 | 2.75E-01 | 2.32E-01 |  |
| OTU113 | 8.29E-02 | -2.38E-01 | -4.81E-01 | 3.18E-01 | 4.08E-01 |  |
| OTU114 | -3.46E-02 | 3.95E-02 | 6.10E-02 | 1.91E-01 | 8.04E-01 |  |
| OTU115 | 2.91E-02 | -9.55E-02 | 8.56E-02 | -1.59E-01 | -1.96E-02 |  |
| OTU116 | 8.46E-03 | -5.17E-02 | -1.09E-01 | 1.50E-01 | -1.62E-01 |  |
| OTU117 | 7.99E-02 | -2.29E-01 | -4.64E-01 | 3.07E-01 | 3.93E-01 |  |
| OTU120 | 4.67E-02 | -1.39E-01 | 2.16E-02 | -1.33E-01 | 1.75E-01 |  |
| OTU121 | -5.36E-03 | -5.32E-02 | -1.05E-01 | 1.04E-01 | -1.73E-01 |  |
| OTU122 | 8.09E-04 | 9.51E-03 | -3.69E-03 | -1.34E-01 | 4.46E-02 |  |
| OTU123 | 3.99E-03 | -7.19E-02 | -1.55E-01 | 1.36E-01 | -1.39E-01 |  |
| OTU126 | 1.37E-02 | -3.61E-02 | 5.94E-02 | -9.84E-02 | 5.95E-02 |  |
| OTU130 | 1.03E-03 | -6.34E-02 | -1.37E-01 | 1.25E-01 | -1.54E-01 |  |
| OTU133 | -2.33E-03 | -1.96E-02 | -2.18E-02 | -1.26E-01 | -1.55E-01 |  |
| OTU135 | -2.06E-02 | -7.57E-02 | 4.67E-03 | 5.57E-02 | -2.17E-01 |  |
| OTU137 | 7.63E-03 | -1.05E-02 | 2.99E-02 | -2.69E-02 | 1.74E-02 |  |
| OTU138 | 6.22E-02 | -1.81E-01 | -3.66E-01 | 2.44E-01 | 3.00E-01 |  |
| OTU146 | 6.71E-03 | -6.51E-02 | -1.39E-01 | 1.18E-01 | -8.78E-02 |  |
| OTU147 | 5.46E-04 | -2.84E-02 | -9.33E-02 | 5.84E-02 | -1.61E-01 |  |
| OTU148 | -3.38E-02 | 7.47E-02 | 2.39E-02 | 2.43E-01 | 7.47E-01 |  |
| OTU157 | 1.07E-02 | -4.59E-02 | -7.13E-02 | 1.01E-01 | -1.20E-01 |  |
| OTU159 | 3.69E-03 | -1.55E-03 | 1.62E-02 | -1.05E-02 | -6.20E-04 |  |
| OTU161 | 1.40E-02 | -2.80E-02 | 2.76E-02 | -4.01E-02 | 4.68E-02 |  |
| OTU162 | 5.14E-03 | -1.36E-02 | 2.23E-02 | -3.69E-02 | 2.23E-02 |  |
| OTU164 | 3.08E-02 | -8.13E-02 | 1.34E-01 | -2.21E-01 | 1.34E-01 |  |
| OTU165 | -3.45E-04 | -4.87E-03 | 1.20E-02 | -4.53E-02 | -5.10E-03 |  |
| OTU166 | 7.44E-04 | -4.56E-02 | -9.90E-02 | 9.00E-02 | -1.11E-01 |  |
| OTU167 | -3.61E-03 | -9.64E-03 | 2.85E-03 | -9.53E-03 | -3.34E-02 |  |
| OTU168 | 2.36E-03 | 1.39E-01 | -1.39E-01 | -9.99E-02 | -5.28E-02 |  |
| OTU169 | -9.70E-03 | -5.53E-02 | -5.16E-02 | 8.06E-02 | -1.61E-01 |  |
| OTU172 | 3.29E-02 | -8.87E-02 | 5.23E-02 | -1.38E-01 | 1.48E-01 |  |
| OTU175 | 4.20E-03 | -1.47E-03 | 1.51E-02 | -2.27E-03 | 2.51E-03 |  |
| OTU176 | 4.49E-02 | -1.28E-01 | -2.26E-01 | 1.35E-01 | 2.19E-01 |  |
| OTU180 | -1.06E-02 | 1.15E-02 | -1.63E-02 | -3.38E-01 | 1.97E-01 |  |
| OTU184 | -8.47E-03 | -1.53E-02 | -3.94E-03 | -9.17E-02 | 7.13E-03 |  |
| OTU187 | 5.14E-03 | -1.36E-02 | 2.23E-02 | -3.69E-02 | 2.23E-02 |  |
| OTU188 | 4.96E-04 | -3.04E-02 | -6.60E-02 | 6.00E-02 | -7.38E-02 |  |
| OTU202 | 1.75E-03 | -7.05E-03 | 1.92E-03 | -7.30E-03 | 1.28E-03 |  |
| OTU204 | -6.45E-03 | -2.37E-02 | 1.46E-03 | 1.74E-02 | -6.79E-02 |  |
| OTU208 | 4.96E-04 | -3.04E-02 | -6.60E-02 | 6.00E-02 | -7.38E-02 |  |
| OTU218 | -2.81E-02 | 9.64E-03 | 8.76E-02 | 2.69E-01 | 6.47E-01 |  |
| OTU233 | 4.13E-05 | -2.54E-03 | -5.50E-03 | 5.00E-03 | -6.15E-03 |  |
| OTU241 | 5.92E-03 | -5.98E-03 | 2.25E-02 | -1.46E-02 | 9.95E-03 |  |
| OTU250 | 1.71E-03 | -4.52E-03 | 7.42E-03 | -1.23E-02 | 7.44E-03 |  |
| OTU259 | -3.66E-02 | 3.43E-03 | 6.74E-02 | 2.72E-01 | 6.60E-01 |  |
| OTU270 | 6.85E-03 | -1.81E-02 | 2.97E-02 | -4.92E-02 | 2.98E-02 |  |
| OTU274 | 1.39E-04 | 8.20E-03 | -8.18E-03 | -5.87E-03 | -3.11E-03 |  |
| OTU286 | 3.43E-03 | -9.04E-03 | 1.48E-02 | -2.46E-02 | 1.49E-02 |  |
| OTU317 | -8.13E-03 | -2.86E-03 | -3.20E-03 | -5.41E-02 | -1.88E-02 |  |
| OTU337 | 3.43E-03 | -9.04E-03 | 1.48E-02 | -2.46E-02 | 1.49E-02 |  |
| OTU342 | -3.45E-02 | 3.36E-02 | 6.48E-02 | 2.72E-01 | 7.63E-01 |  |
| OTU346 | -6.63E-04 | 7.17E-04 | -1.02E-03 | -2.11E-02 | 1.23E-02 |  |
| OTU354 | 1.71E-03 | -4.52E-03 | 7.42E-03 | -1.23E-02 | 7.44E-03 |  |
| OTU362 | 2.49E-03 | 3.05E-03 | 7.65E-03 | 1.00E-02 | -4.92E-03 |  |
| OTU469 | -6.90E-02 | 6.48E-02 | 1.24E-01 | 5.49E-01 | 1.52E+00 |  |
| OTU665 | -1.04E-01 | 1.01E-01 | 1.94E-01 | 8.16E-01 | 2.29E+00 |  |
|  |  |  |  |  |  |  |
|  |  |  |  |  |  |  |
| Site scores (weighted sums of species scores) | | | |  |  |  |
|  |  |  |  |  |  |  |
|  | RDA1 | RDA2 | RDA3 | RDA4 | RDA5 |  |
| C_day3 | -0.34467 | 1.97135 | -0.1898 | 0.36142 | 0.1628 |  |
| C_day14 | -1.10319 | -0.11432 | -0.1497 | -0.81017 | 0.4088 |  |
| C_day17 | -3.02207 | -0.83616 | 0.2943 | 0.04006 | 0.1997 |  |
| C_day21 | -2.62991 | 0.23032 | 0.3551 | 0.40791 | 0.4739 |  |
| C-day28 | 2.08829 | -0.09948 | -1.0968 | -0.07146 | 0.3609 |  |
| C-day42 | 3.42316 | 0.08832 | 1.08 | 0.32611 | -0.1274 |  |
| T-pl_day3 | -2.39899 | 0.436 | 0.2524 | 0.33223 | -0.3924 |  |
| T-pl_day14 | 0.07254 | 0.22281 | -0.4675 | -0.5303 | -0.4926 |  |
| T-pl_day17 | -3.37297 | -0.7041 | 0.4802 | 0.27955 | -0.7403 |  |
| T-pl_day21 | 1.90401 | -0.23089 | -0.3079 | -0.44247 | -0.3219 |  |
| T-pl_day28 | 2.22462 | -0.59042 | -1.1056 | 0.47512 | 0.2297 |  |
| T-pl_day42 | 3.15918 | -0.37343 | 0.8553 | -0.368 | 0.2388 |  |
|  |  |  |  |  |  |  |
|  |  |  |  |  |  |  |
| Site constraints (linear combinations of constraining variables) | | | | |  |  |
|  |  |  |  |  |  |  |
|  | RDA1 | RDA2 | RDA3 | RDA4 | RDA5 |  |
| C_day3 | -0.49468 | 1.46854 | -0.12455 | 0.0793 | -0.17599 |  |
| C_day14 | -1.11083 | 0.09977 | -0.12538 | -0.731744 | 0.18673 |  |
| C_day17 | -3.2554 | -0.56795 | 0.30536 | 0.161569 | -0.24683 |  |
| C_day21 | -2.72704 | 0.22071 | 0.37528 | 0.444589 | 0.54671 |  |
| C-day28 | 0.05118 | -0.26061 | -0.49912 | 0.12808 | -0.0691 |  |
| C-day42 | 4.06836 | 0.4138 | 0.91544 | 0.33877 | -0.07294 |  |
| T-pl_day3 | -0.74075 | -0.01051 | 0.11958 | -0.245586 | -0.04092 |  |
| T-pl_day14 | 0.1689 | 0.82786 | -0.72949 | -0.147717 | -0.03427 |  |
| T-pl_day17 | -2.26689 | -0.68995 | 0.03759 | 0.12651 | -0.21633 |  |
| T-pl_day21 | 0.69034 | -0.21517 | 0.18039 | -0.006405 | -0.11395 |  |
| T-pl_day28 | 3.01308 | -0.71643 | -1.28194 | 0.239186 | 0.13434 |  |
| T-pl_day42 | 2.60374 | -0.57007 | 0.82683 | -0.386552 | 0.10253 |  |
|  |  |  |  |  |  |  |
|  |  |  |  |  |  |  |
| Biplot scores for constraining variables | | |  |  |  |  |
|  |  |  |  |  |  |  |
|  | RDA1 | RDA2 | RDA3 | RDA4 | RDA5 |  |
| pH | -0.6438 | 0.56315 | 0.4086 | -0.3185 | -0.001504 |  |
| DCW | -0.6489 | 0.09805 | 0.2053 | 0.391 | 0.611802 |  |
| TDN | 0.8823 | -0.3733 | 0.1942 | 0.2106 | 0.010934 |  |
| TDP | -0.4634 | 0.61793 | 0.1669 | 0.4466 | -0.419637 |  |
| Chl-a | -0.5246 | 0.15512 | 0.1754 | 0.3665 | 0.731889 |  |
